# Histological and histomorphometric evaluation of alveolar ridge preservation using an allograft and nonresorbable membrane with and without primary closure: A pilot randomized controlled clinical trial

**DOI:** 10.1097/MD.0000000000029769

**Published:** 2022-06-30

**Authors:** Majdi A. Aladmawy, Zuhair S. Natto, Michael Kreitzer, Yumi Ogata, Yong Hur

**Affiliations:** a Department of Periodontology, Johns Hopkins Aramco Healthcare, Dhahran, Saudi Arabia; b Department of Periodontology, Tufts University School of Dental Medicine, Boston, MA; c Department of Dental Public Health, Faculty of Dentistry, King Abdulaziz University, Jeddah, Saudi Arabia; d Private practice, Dublin, OH.

**Keywords:** alveolar ridge preservation, bone allograft, closed flap, histomorphometric analysis, nonresorbable membrane, open flap

## Abstract

The aim of this article is to assess the formation of new vital bone (VB) using histomorphometric analysis in alveolar ridge preservation (ARP), with and without primary closure. Eight patients needed bilateral tooth extraction and planned for ARP. All patients had a nonresorbable membrane with freeze-dried bone allograft after the extractions. Biopsies were obtained 6 months after ARP and were evaluated using histomorphometric analysis. The study included 6 males and 2 females, with an average age of 54.2 years (standard deviation, 9.7). The teeth requiring extraction included a bilateral canine (1 case), premolars (5 cases), and molars (2 cases). Histomorphometric values of new VB, residual bone (RB) substitute particles, and marrow tissue formation were 71.1 %, 16.2%, and 9.69% for closed flap and 50.9%, 15.3%, and 8.19 for open flap. *P* values were 0.066, 0.878, and 0.326, respectively. The present findings indicate that leaving the flap without primary closure did not have any effect on new VB, RB particles, and immature bone marrow compared with closed flap. However, the results favored the closed-flap technique.

## 1. Introduction

Alveolar ridge preservation (ARP) procedures have been proven to minimize the amount of ridge shrinkage, with several studies having demonstrated the beneficial results of ARP in reducing the amount of ridge shrinkage compared with socket healing without any ARP procedure.^[1–4]^ However, there is extensive variation in terms of ARP techniques, measurements methods, evaluation time, and materials used.^[5,6]^

There are several methods of ARP.^[7,8]^ Several articles view ARP as a type of guided bone regeneration in which a primary wound closure is required.^[5,6]^ It is important to have a primary closure to regenerate the maximum amount of bone.^[5,9]^ We may have insufficient bone formation if the membrane is exposed.^[5,9]^ However, it is difficult to have primary closure in some cases, and some clinicians may adopt the controversial method of leaving the membrane exposed.^[5,9]^ Type of membrane is a critical factor in the decision to leave the membrane exposed (open flap technique [OFT]) or not (closed-flap technique [CFT]).^[10]^ The exposure of expanded polytetrafluoroethylene (e-PTFE) membrane contains a high risk of infection and interrupts bone formation.^[10]^ However, collagen membrane and a dense polytetrafluoroethylene (d-PTFE) membrane have been found to contain less potential risk when exposed and do not inhibit bone formation.^[8,11–13]^

Moreover, the type of biomaterial used such as xenografts or allografts may play a role in ARP.^[14]^ The type of bone graft selected for use may differ depending on clinician preference, cost, or cultural background. Freeze-dried bone allograft (FDBA) is one of the most commonly used materials used for ARP.^[2]^ It acts as a scaffold, increases bone vitality, and maintains space.^[15,16]^ A recent article has shown that using an FDBA in combination with a membrane exhibits a smaller amount of bone height reduction compared with other treatment options.^[2]^ However, the effect of open flap (OF) versus closed flap (CF) on FDBA remains uncertain. Additionally, it is difficult to apply CF in many daily cases, due to local conditions such as buccolingual dimension of the socket and quality of the surrounding soft tissue. Therefore, this article aims to assess the formation of new bone using histomorphic analysis.

## 2. Materials and Methods

**Figure 1.**
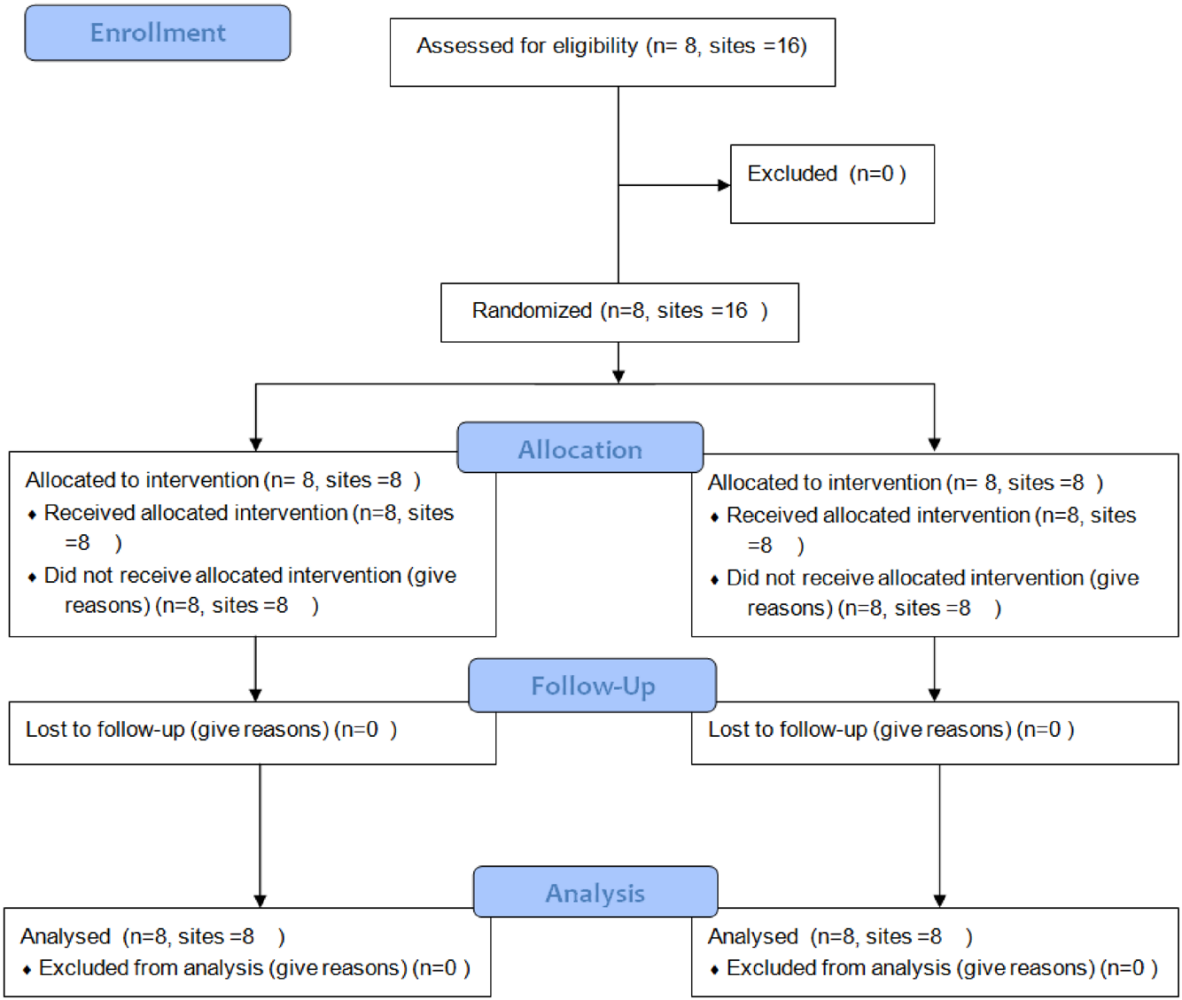
Consolidated Standards of Reporting Trials (CONSORT) flow diagram.

This was a pilot split mouth two-arm randomized clinical trial held at Tufts University School of Dental Medicine, with Institutional Review Board number 11441. The patients were recruited, and ARP was conducted between January 2016 and January 2017. The study was registered at ClinicalTrials.gov (NCT03136913), and the Consolidated Standards of Reporting Trials guideline was followed (Fig. 1). The clinical outcomes of this study were published elsewhere.^[10]^

### 2.1. Participants

The study included patients with the following criteria: need a bilateral extraction of any posterior teeth or canine within the same arch and consequently require ARP procedure, at least 18 years old, no medical contraindications, three wall bony defect, not pregnant or smoking, and possessing optimal oral hygiene (plaque score of <15% and bleeding score of <10%).

### 2.2. Surgical procedure

**Figure 2.**
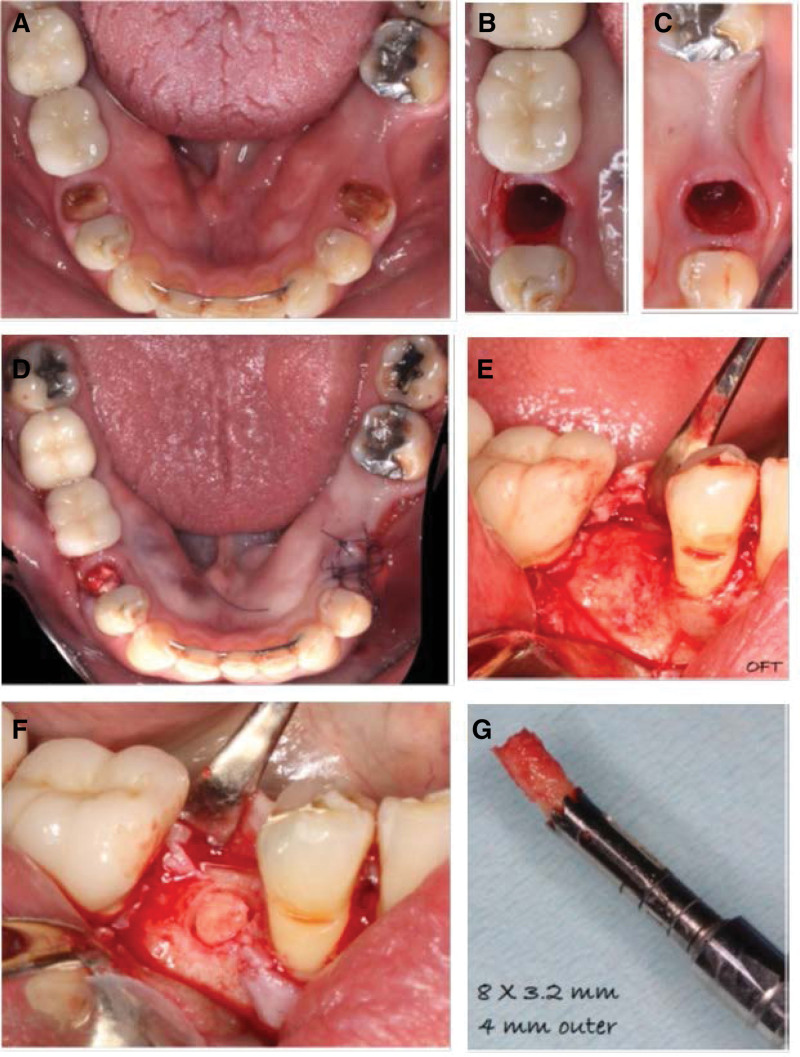
Surgical steps. (A) Patient needed bilateral extraction. (B, C) Atraumatic bilateral extraction. (D) Placed bone graft then membrane was exposed in one side and closed in other side. (E) Flap reflection after 6 mo. (F) Implant area was prepared for a biopsy. (G) Core biopsy was obtained.

The randomization of each side to receive either primary closure (CF) or no closure (OF) was generated using a computer program statistical package (R Version 2.11.1, R Foundation for Statistical Computing, Vienna, Austria) (Fig. 2A). Treatment options were concealed, and patients were allocated to the treatment at the day of the surgery. Atraumatic extraction was performed for all teeth (Fig. 2B and C). Sectioning was required for molar teeth to facilitate the extraction.

A trapezoidal flap was conducted in the CF group. A full thickness flap was reflected up to the mucogingival junction, followed by a split thickness flap apically. Next, an allograft FDBA (MinerOss, BioHorizons, Birmingham, AL, USA) was placed after hydration in adherence to the manufacturer’s instructions. A d-PTFE nonresorbable membrane (Cytoplast TXT-200, Osteogenics Biomedical, Lubbock, TX, USA) was cropped to the desired size and extended by 3 mm apical to the flap margins. Finally, flaps were secured by sutures to cover the membrane with primary closure (VICRYL, polyglactin 910, Ethicon, Somerville, NJ, USA) (Fig. 2D).

In the OF group, intrasulcular incisions were used exclusively and included in the buccal and lingual sides adjacent to at least 1 tooth. All the other steps were identical to those in the CF group except the final step, in which the flaps were repositioned, and d-PTFE were left exposed without any primary closure (Fig. 2D). After the ARP procedures, patients in both groups received the following: 500 mg of amoxicillin (3 times per day), 0.12% of chlorhexidine, and 600 mg of ibuprofen (3 times per day).

### 2.3. Histological and histomorphometric analysis

The obtained bone specimens were collected 6 months after ARP during the implant placement procedure, and the membrane was removed at the time of implant placement. The specimens were collected using a trephine bur and fixed in 10% buffered formalin (Fig. 2E and F). Each bone core was then decalcified in 5% formic acid and socked in paraffin. Several longitudinal sections (4–6-μm thickness) were conducted through the middle of each specimen using a microtome. The first section was stained with hematoxylin and eosin and the subsequent sections with Masson trichrome. A histomorphometric investigation was conducted using appropriate software (PhotoshopCS6, Adobe, CA). The percentages of newly vital bone (VB), residual bone (RB) graft, and marrow tissue (MT) formation were assessed.

### 2.4. Sample size and statistical analysis

This is a pilot study, and no formal sample size calculation was performed. Eight subjects were included with a total of 16 sites and were analyzed as per protocol. Descriptive statistics (e.g., means and standard deviations [SDs]) were calculated for each group. The primary outcome was bone vitality, and secondary outcomes were RB graft and immature bone. The paired *t* test was used for statistical analysis after checking the normality distribution. *P* values of <0.05 were statistically significant. Statistical Package for the Social Sciences (SPSS) Version 23 (IBM Inc., Armonk, NY) was used in the analysis.

## 3. Results

### 3.1. Demographics

The participants included 6 men and 2 women. Their average age was 54.2 years (SD, 9.7). The study included bilateral canine (1 case), premolar (5 cases), and molar (2 cases) teeth in need of extraction and ARP (Table 1).

**Table 1:** Summary of demographic data.

Variable	N (%)
Age (mean ± SD)	54.2 ± 9.7
Gender	
Males	6 (75.0)
Females	2 (25)
Teeth position	
Bilateral premolars	5 (62.5)
Bilateral molars	2 (25.0)
Bilateral canine	1 (12.5)

SD = standard deviation.

### 3.2. Bone core biopsy

**Figure 3.**
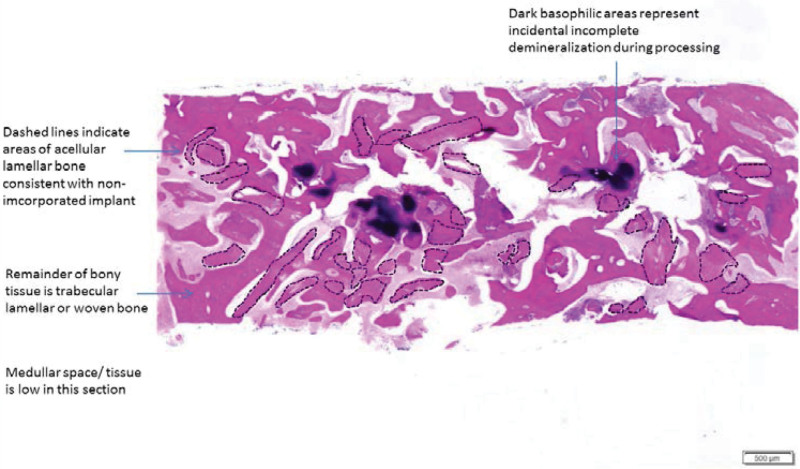
Panoramic histological section of bone core biopsy taken after 6 mo with primary closure.

**Figure 4.**
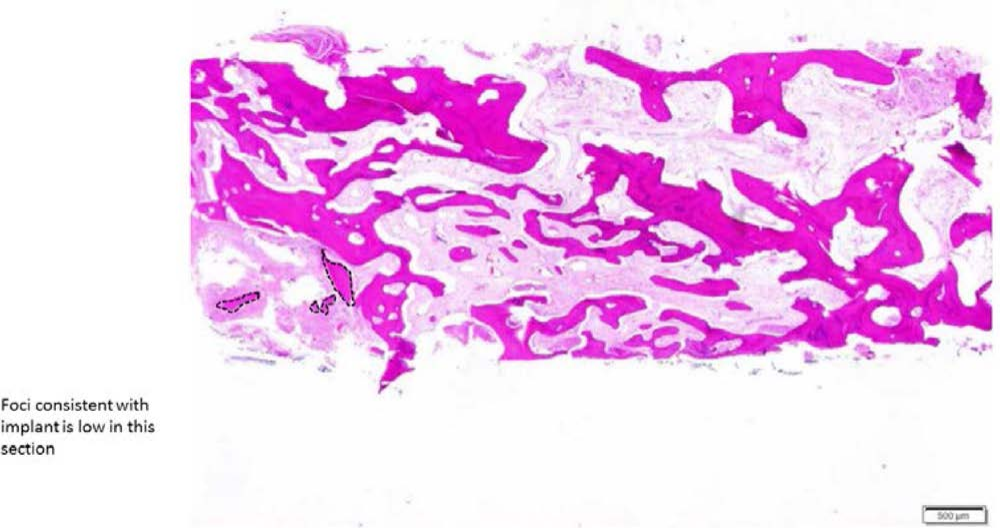
Panoramic histological section of bone core biopsy taken after 6 mo without primary closure.

**Figure 5.**
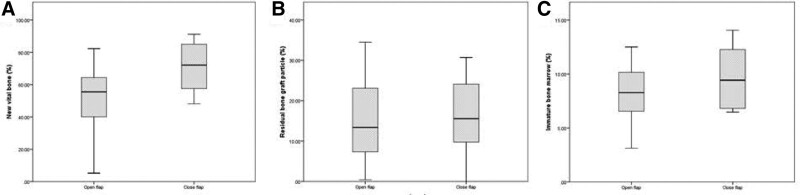
Box plot diagram of the percentage of (A) new vital bone, (B) residual bone of the particle, and (C) immature bone marrow.

Biopsies were obtained from all 16 sites. Newly VB was formed in close contact with RB substitute particles. Low amount of MT formation was reported in both groups (Figs. 3 and 4). Histomorphometric values of VB, RB, and MT were 71.1 %, 16.2%, and 9.69% for CF and 50.9%, 15.3%, and 8.19% for OF, respectively (Fig. 5). *P* values were 0.066, 0.878, and 0.326, respectively (Table 2).

**Table 2:** Summary of the histomorphic analysis.

Variable	Total	Open	Closed	*P* value
New vital bone	61.0 ± 22.1	50.9 ± 16.2	71.1 ± 23.5	0.066
Residual bone graft particle	15.8 ± 10.4	15.3 ± 10.1	16.2 ± 11.4	0.878
Immature bone marrow	8.94 ± 2.94	8.19 ± 2.94	9.69 ± 2.93	0.326

*P* value < 0.05.

## 4. Discussion

The current study assessed the effects of using d-PTFE membrane and FDBA on ARP with OF compared with CF. The subsequent results demonstrate that CF has more new VB and bone marrow and less residual graft particles compared with OF, which again indicates the potential effect of primary closure on ARP procedures.

Previous studies have revealed that there is no difference between CF and OF regarding bone height and width. However, regarding the width of keratinized tissue, OF is generally favored over CF, whereas it was vice versa regarding the level of pain and discomfort involved.^[5,10]^ Several studies have recommended the use of d-PTFE membrane in ARP of soft and hard tissues^[17–19]^ due to the low porosity (<0.3 μm). This characteristic makes this membrane more resistant to bacterial infiltration during membrane exposure.^[20]^ Moreover, there are several clinical, radiographic, and histological advantages to d-PTFE membrane compared with e-PTFE membrane;^[11,21,22]^ however, the evidence remains insufficient.^[11,21,22]^ In addition, the use of this membrane contains the disadvantage of requiring an additional interventional step, namely membrane removal.^[21]^

Moreover, using d-PTFE membrane in ARP results in dimensional changes between −0.3 and −3.8 mm horizontally and between −1.31 and +0.45 vertically.^[11,19,21]^ This is also comparable with other membranes used in ARP, which produce bone loss ranging from 1.99 to 2.37 mm in width and from 1.10 to 1.72 mm in height.^[3]^ This could be related to differences in surgical techniques, bone graft materials, and assessment methods.

Another factor that may play a significant role is the quality of the tissue below the d-PTFE membrane, which appeared as reddish and friable and contained a dense connective matrix, fibroblasts, and inflammatory cells without epithelial cells.^[21]^ Tissues may act as a temporary matrix for epithelialization. This avoids the invasion of oral environment factors to the bone graft material. In another study, epithelialization was successfully formed in all cases without the exposure of any bone graft particles.^[5]^

The average amount of newly vital formed bone in these biopsies was 71.1% ± 23.5% in the CF and 50.9 ± 16.2 in the OF (*P* = 0.066). Systematic review and meta-analysis studies have found a comparable amount of new bone formation in sockets grafted with FDBA, which ranged between (mean, 67; 95% CI, 32.09–91.80%) at 7 months of follow-up.^[14]^ Moreover, FDBA has the lowest RB graft materials compared with types of bone graft (12.4–21.11%) and is the highest among xenografts and alloplasts. The variation in the results may be attributed to several factors, such as the number of sockets’ walls, socket size, amount of bone loss, direction of the core biopsy, and the number of participating patients.

One of the current study’s limitations is the use of convenience sampling. However, this is a pilot study and represents results relevant to daily clinical practice. Additionally, the study does not include any information about potential confounders such as defect size and the condition of walls, which can be addressed in future studies.

In conclusion, the present findings demonstrate that leaving the flap open does not have any effect on new VB, RB particles, or immature bone marrow compared with CF. However, the results favor CFT, indicating that primary closure might be a critical factor for ARP in new bone vitality.

### Author contributions

All authors have read and agreed to the published version of the manuscript. Conceptualization: M.A., Z.N., Y.O., and Y.H.; methodology: M.A., Z.N., and M.K.; software: M.A. and Z.N.; validation: M.A. and M.K.; formal analysis: Z.N.; investigation: M.A. and M.K.; resources: M.A.; data curation: M.A., Z.N., and M.K.; writing—original draft preparation: M.A. and Z.N.; writing—review and editing: M.A., Z.N., Y.O., and Y.H.; supervision: Y.O. and Y.H.; project administration: M.A., Y.O., and Y.H.; funding acquisition: M.A.
